# Global Responses of Il-1β-Primed 3D Tendon Constructs to Treatment with Pulsed Electromagnetic Fields

**DOI:** 10.3390/cells8050399

**Published:** 2019-04-30

**Authors:** Renate Gehwolf, Bettina Schwemberger, Malik Jessen, Stefanie Korntner, Andrea Wagner, Christine Lehner, Nadja Weissenbacher, Herbert Tempfer, Andreas Traweger

**Affiliations:** 1 Institute of Tendon and Bone Regeneration, Paracelsus Medical University—Spinal Cord Injury & Tissue Regeneration Center Salzburg, 5020 Salzburg, Austria; renate.gehwolf@pmu.ac.at (R.G.); b.schwemberger@pmu.ac.at (B.S.); m.jessen@stud.uni-heidelberg.de (M.J.); andrea.wagner@pmu.ac.at (A.W.); christine.lehner@pmu.ac.at (C.L.); nadja.weissenbacher@pmu.ac.at (N.W.); herbert.tempfer@pmu.ac.at (H.T.); 2 Austrian Cluster for Tissue Regeneration, 1200 Vienna, Austria; 3 Institute of Transfusion Medicine and Immunology, Medical Faculty Mannheim, German Red Cross Blood Donor Service Baden-Württemberg-Hessen gGmbH, Heidelberg University, 68167 Mannheim, Germany; 4 Regenerative, Modular & Developmental Engineering Laboratory (REMODEL); Science Foundation Ireland Centre for Research in Medical Devices (CÚRAM) National University of Ireland Galway; H91 W2TY Galway, Ireland; stefanie.korntner@nuigalway.ie

**Keywords:** tendon, tendinopathy, pulsed electromagnetic field (PEMF), repetitive peripheral magnetic stimulation (rPMS), Il-1β, decoy receptor Il1r2, RNA-Seq, apoptosis

## Abstract

Tendinopathy is accompanied by a cascade of inflammatory events promoting tendon degeneration. Among various cytokines, interleukin-1β plays a central role in driving catabolic processes, ultimately resulting in the activation of matrix metalloproteinases and a diminished collagen synthesis, both of which promote tendon extracellular matrix degradation. Pulsed electromagnetic field (PEMF) therapy is often used for pain management, osteoarthritis, and delayed wound healing. In vitro PEMF treatment of tendon-derived cells was shown to modulate pro-inflammatory cytokines, potentially limiting their catabolic effects. However, our understanding of the underlying cellular and molecular mechanisms remains limited. We therefore investigated the transcriptome-wide responses of Il-1β-primed rat Achilles tendon cell-derived 3D tendon-like constructs to high-energy PEMF treatment. RNASeq analysis and gene ontology assignment revealed various biological processes to be affected by PEMF, including extracellular matrix remodeling and negative regulation of apoptosis. Further, we show that members of the cytoprotective Il-6/gp130 family and the Il-1β decoy receptor Il1r2 are positively regulated upon PEMF exposure. In conclusion, our results provide fundamental mechanistic insight into the cellular and molecular mode of action of PEMF on tendon cells and can help to optimize treatment protocols for the non-invasive therapy of tendinopathies.

## 1. Introduction

Tendon overuse injuries and tendinopathies represent debilitating conditions affecting both the working population and recreational athletes, and represent one of the most frequent musculoskeletal conditions for which patients seek medical advice [1]. Despite many medical advances, acute tendon injuries and chronic tendinopathies remain clinically challenging [2]. The hypocellular and hypovascular nature in combination with the intricate architecture of the extracellular matrix (ECM) significantly impedes tissue healing, resulting in biomechanically inferior scar tissue prone to re-rupture and often favoring the development and progression of degenerative processes [3]. Generally, outcomes from current treatments, including physiotherapy, ultrasound, extracorporeal shockwave therapy, non-steroidal anti-inflammatory drugs, and ultimately surgery are often unsatisfactory and rehabilitation times can be extensive [4].

Development of effective treatment strategies is currently hampered by our poor understanding of the molecular and cellular events underlying tendinopathy. In the past tendinopathy has primarily been considered as a non-inflammatory, degenerative process. Indeed, there is an ongoing debate on whether inflammation is a key driver of tendinopathy. However, recent studies convincingly demonstrate that a cascade of inflammatory events, such as lymphocyte and macrophage infiltration, matrix metalloprotease (MMP) activation, and secretion of inflammatory mediators, including various cytokines, prostaglandins, and nitric oxide [5,6,7] are central to the etiology of tendinopathies. Although the key cytokines in tendon disease have not yet been fully defined, family members of the interleukin-1 (Il-1) family, in particular Il-1β, can trigger catabolic degradation of the ECM via the activation of MMPs, thereby contributing to the progression of tendinopathy [8,9]. Therefore, targeting the inflammatory cascade to shift a “degenerative” to a “regenerative” inflammatory response and reducing aberrant ECM remodeling seems promising to manage tendinopathies.

Several conservative treatment regimens are being applied to reduce pain and improve tendon function, including various electrotherapy modalities. One emerging therapy for the treatment of both acute and chronic inflammation is the application of low and high energy pulsed electromagnetic fields (PEMF) [10,11], the latter also termed repetitive peripheral magnetic stimulation (rPMS). In 1979, the U.S. Food and Drug Administration (FDA) approved PEMF as safe and effective to treat delayed and non-unions in bone [12]. Since then, PEMF has been proposed to be effective in treating a variety of pathologies, including delayed wound healing [13], chronic postoperative pain [14], and osteoarthritis [15,16]. In vitro, PEMF has been shown to be potent in limiting the catabolic effects of pro-inflammatory cytokines on hyaline cartilage [17,18], suggesting that PEMF is able to limit inflammation and promote soft tissue repair. In addition, low intensity PEMF has been demonstrated to inhibit TNF-α- or LPS-induced PGE2 release in bovine synovial fibroblasts, most likely by modulation of adenosine-mediated anti-inflammatory pathways [19]. More recently, the feasibility to apply PEMF for the management of tendon disorders has been investigated. PEMF-treatment of human tenocytes in 2D in vitro cell cultures resulted in an altered cytokine and TGFβ release profile and an increased expression of type I collagen [20]. In vivo preclinical studies also indicate that treatment with a low frequency pulsed electromagnetic field can improve rat Achilles tendon repair [21,22]. Finally, in a randomized, controlled clinical study Osti et al. demonstrated that PEMF-treatment reduced postoperative pain, analgesic use, and stiffness after rotator cuff repair. Interestingly, the authors did however not observe any functional improvement in a 2-year follow-up [23]. 

Overall, our understanding of the biologic responses of tenocytes to a physical signal from an electromagnetic stimulus remains very limited. Therefore, the objective of this study was to investigate the transcriptome-wide responses of 3D tendon-like constructs cultured under pro-inflammatory conditions to high energy PEMF/rPMS treatment. The results of this study provide mechanistic insights into the cellular and molecular ramifications of PEMF treatment and will support the development of optimized protocols for the non-invasive treatment of tendinopathies.

## 2. Materials and Methods

### 2.1. Animals and Cell Culture

Three months old female Fischer344 rats were used for all experiments. Tissue donor rats were housed and euthanized in accordance with the respective Austrian laws on animal welfare and experimentation. 

Tendon stem and progenitor cells (subsequently referred to as TDSPCs) were isolated and cultured as previously described [24]. Briefly, Achilles tendons were dissected under sterile conditions and washed with sterile PBS without Ca^2+^ and Mg^2+^ (subsequently referred to as PBS) and α-MEM (minimal essential medium with 2 mM GlutaMAX^TM^). Tendon tissue was then cut into small pieces and incubated in 3 mg/mL Collagenase Type II (Gibco Lifetechnologies, Vienna, Austria) in α-MEM with 10% fetal bovine serum (FBS), 2 mM Glutamax, 100 units mL^−1^ penicillin and 0.1mg mL^−1^ streptomycin (P/S, Sigma-Aldrich, Vienna, Austria), O/N at 37 °C, 5% CO_2_, and 90% humidity. The following day, cells were washed in α-MEM with 10% FBS, 2 mM Glutamax, and P/S and cultivated until near confluency. Cell number and cell viability were determined with a LunaTM-fl cell counting system (Logos Biosystems, Annandale, VA, USA) according to the manufacturer’s instructions. All experiments were performed with cells at passage 3. 

### 2.2. In Vitro Tendon-Like Construct Formation

3. D-tendon constructs were assembled as described in reference [25]. In brief, each petri dish was coated with 15 mL of Sylgard 184 silicone elastomer (Dow-Chemicals, Vienna, Austria) and allowed to cure at 48 °C O/N. Subsequently, 2 × 0.8 mm long silk sutures were pinned with minutien insect pins (0.1 mm diameter, Science Service, Munich, Germany) onto the silicone layer with 1cm distance between the two sutures (8 units per culture dish). Plates were then sterilized with 70% ethanol, and exposed to UV-irradiation for 30 min each. Before use plates were washed with sterile PBS. TDSPCs (passage 3) were re-suspended in 2 mg/mL ice cold PureCol Collagen type 1 (Sigma-Aldrich, Vienna, Austria) in α-MEM without serum supplemented with P/S to a final cell number of 2.5 × 10^5^ mL^−1^. Cell suspension (130 µL for each construct) was then immediately pipetted between the 2 silk sutures. Collagen was then allowed to polymerize for 1h at 37 °C in a cell culture incubator. Finally, 15 mL of complete culture medium (α-MEM supplemented with 10% FBS, 2 mM GlutaMax, P/S, 10 µg/mL aprotinin, 0.2 mM ascorbic acid, and 0.05 mM l-proline) was added and the medium was exchanged every other day. After 7 days of contraction the tendon-like constructs were used for PEMF treatment.

### 2.3. Pro-Inflammatory Stimulation/Conditioning of Tendon-Like Constructs

Pro-inflammatory stimulation of tendon-like constructs was done after 7 days of contraction with 10 ng/mL recombinant rat interleukin-1β (rIl-1β; PeproTech, Vienna, Austria) in complete culture medium for 24 h. One hour before PEMF treatment/exposure rIl-1β containing medium was replaced with complete culture medium w/o rIl-1β. Supernatants were collected before rIl-1β treatment (supernatant 1), after 24 h of rIl-1β incubation (supernatant 2) and after the completed PEMF exposure protocol (2 cycles of 60 min PEMF exposure and 90 min resting time each; supernatant 3) and stored at −80 °C until further use. 

### 2.4. PEMF Treatment

High energy PEMF/rPMS treatment was performed with an OMNITRON promed^©^ device (Healthfactories GmbH, Surheim, Germany). The maximum magnetic flux density and fundamental frequency of the PEMF signal were 82 mT and 125 kHz, respectively. Pulse frequency was 2 Hz with a burst duration of 80 µsec. 3D tendon constructs were treated as follows: 2 treatment cycles at 82 mT for 60 min each followed by 90 min resting time were performed in a cell culture incubator at 37 °C, 5% CO_2_, and 90% humidity. Constructs were harvested 90 min after the second exposure. 

### 2.5. Cell Viability, Metabolic Activity, and Cytotoxicity Assays

Cell viability was analyzed by LIVE/DEAD imaging kit (Invitrogen, Vienna, Austria) according to manufacturer´s instructions on a LSM700 Laser scanning microscope (Carl Zeiss, Jena, Germany). Cell metabolic activity and cytotoxicity after PEMF treatment were determined using CellTiter 96^®^ AQueous One Solution Cell Proliferation Assay (MTS) and by measuring the total ATP content using the CellTiter Glo^®^ 2.0 Assay (both Promega, Vienna, Austria) following the manufacturer´s instructions.

### 2.6. RNA Isolation, Quantification and Validation

Tendon-like constructs were washed in sterile PBS and then homogenized in TRIZol (FisherScientific, Vienna, Austria) on ice using an Ultra-Turrax (IKA, Staufen, Germany). Total RNA was prepared according to manufacturer´s instructions with minor modifications. Two additional chloroform extraction steps were performed. Subsequently, total RNA was precipitated for 30 min at −20 °C with an equal volume of ice cold isopropanol and by the addition of 1µg GlycoBlue co-precipitant (Ambion, Lifetechnologies, Vienna, Austria), followed by centrifugation for 30 min at 13,000 rpm at 4 °C. RNA pellets were resuspended in RNase-free water supplemented with 20 units of Superase^TM^ RNase inhibitor (Ambion, Lifetechnologies, Vienna, Austria) and stored at −80°C until further use. RNA yield was quantified using a Nanodrop 2000C (ThermoFisher Scientific, Vienna, Austria) and RNA integrity was verified using an Experion Automated Electrophoresis system (Biorad, Munich, Germany). A minimum requirement of RNA quality indicator (RQI) >9.0 was chosen for RNA sequencing and RT-qPCR.

### 2.7. RNASeq and Data Analyses

Library preparation and RNA Sequencing (mRNA sequencing, 50bp, 30M single-end reads per sample) were performed at Exiqon (Qiagen, Hilden, Germany). Analysis was performed on tendon constructs established with tendon cells isolated from 3 individual rats. Differential expression and gene ontology (GO) term enrichment was performed using FunRich (http://www.funrich.org/; v 3.1.3; GO database Norway rat, ID:10116). Genes with an adjusted p-value of less than 0.05 were used for further analysis and candidate genes were verified by quantitative RT-PCR.

### 2.8. Reverse Transcription and Gene Expression Analysis

First strand cDNA synthesis was performed with iScript Supermix^TM^ (Biorad, Munich, Germany) according to manufacturer´s recommendations. For quantitative PCR 5 ng/well cDNA was subsequently analyzed using TaqMan Assays (either from Integrated DNA Technologies or Applied Biosystems, see supplementary Table S4) and Luna^®^ Universal Probe qPCR Master Mix (New England Biolabs, Frankfurt am Main, Germany). Amplification conditions were as follows: initial denaturation for 60 s at 95 °C, followed by 40 cycles of 15 s at 95 °C and 30 s at 60 °C. All samples were run in duplicates and CQ values were analyzed using qBaseplus v2.4 (Biogazelle) and normalized relative quantities were calculated by normalizing the data to the expression of previously validated endogenous control genes as described in reference [26]. 

### 2.9. Protein Lysates, SDS-PAGE and Western Blot

Tendon-like constructs were washed in PBS, lysed and homogenized in ice cold RIPA buffer (Sigma-Aldrich, Vienna, Austria) supplemented with protease inhibitor cocktail (Sigma-Aldrich, Vienna, Austria; with final concentrations of AEBSF (4-(2-Aminoethyl)benzensulfonylfluorid) at 1.04 mM, Aprotinin at 0.8 µM, Bestatin at 40 µM, Leupeptin at 20 µM, E-64 at 14 µM, and Pepstatin A at 15 µM) and 1× phosphatase inhibitor cocktail 3 (Sigma Aldrich, Vienna, Austria). 10 to 20 µg of total protein was separated on 10–12% TGX Stain-free^TM^ polyacrylamide gels (Biorad, Munich, Germany) in Laemmli buffer [27]. Proteins were then transferred to a polyvinylidene fluoride (PVDF) membrane (Biorad, Munich, Germany) and subsequently membranes were blocked for 2 h in 5% BSA in Tris buffered saline with 0.05% Tween 20 (subsequently referred to as TBST). Membranes were probed O/N at 4 °C in primary antibodies (mouse ani-ERK1/2; 1:1000; R&D Systems and rabbit anti-phospho-p44/42 ERK1/2 (Thr202/Tyr204), 1:2000; Cell Signaling Technologies) in a blocking solution. After washing membranes in TBST, membranes were probed with appropriate peroxidase-conjugated secondary antibodies in TBST for 1h at RT. After final washing, blots were developed using a Chemidoc MP Imaging System and the Clarity Western ECL substrate (Biorad, Munich, Germany).

### 2.10. Cryo-Embedding and Sectioning

Tendon-like constructs were washed in PBS and fixed in 4% paraformaldehyde for 1h at RT, washed twice for 15 min in PBS, incubated serially O/N in 15% (*w*/*v*) sucrose in PBS, O/N in 30% (*w*/*v*) sucrose in PBS, and O/N in a 1:1 mixture of 30% sucrose and Surgipath^®^ FSC22^®^ Frozen section compound (Leica Microsystems, Vienna, Austria). Finally, constructs were cryoembedded in Surgipath^®^ FSC22^®^ Frozen section compound on dry ice and stored at −80 °C until further use. Sectioning (10 and 20 µm sections) was done with a CM1950 Cryostat microtome (Leica Microsystems, Vienna, Austria). 

### 2.11. TUNEL Staining and Caspase3/7 Activity Assay

Determination of apoptosis was performed by staining of cryosections with the In Situ Cell Death Detection Kit, Fluorescein (Roche, Vienna, Austria) according to the manufacturer´s recommendations. Quantification of TUNEL staining was performed using ImageJ software ImageJ software v. 150a [28]. Caspase3/7 activity was measured using protein lysates of tendon-like constructs and the Caspase-Glo^®^ 3/7 Assay (Promega, Vienna, Austria). 

### 2.12. Quantification of Nuclear Aspect Ratio, Cell Orientation and Collagen Density

Polarization microscopy was used for the determination of the type I collagen fiber orientation and packing density. Birefringence intensity was measured by quantification of average pixel intensity using ImageJ software v. 150a [28]. For polarization microscopy, unstained sections of tendon-like constructs were imaged using a 10× or 16× objective equipped with a polarization filter mounted on an Axioplan microscope (Carl Zeiss, Jena, Germany). Cell orientation was determined by calculation of the nuclear aspect ratio [29] and the angular deviation of stress fiber orientation on sections stained with rhodamine-phalloidin. 

### 2.13. Interleukin-6 ELISA and Quantification of NO Production

Interleukin-6 (Il-6) secretion of tendon-like constructs was determined in cell culture supernatants collected as described above using a rat Il-6 ELISA kit (RayBiotech, Norcross, GA, USA) according to the manufacturer´s instructions. 

The detection of NO metabolites in cell culture supernatants was performed with a colorimetric Nitrite/Nitrate Assay Kit (Sigma-Aldrich, Vienna, Austria) according to the manufacturer’s instructions. For determination of nitrate concentration, 5 µL nitrate reductase and 5 µL enzyme co-factor solution were added to 40 µL sample in a 96 well plate. After an incubation of 2 h at 25 °C with shaking, 50 µL Griess Solution A was added to each well and incubated for 15 min at 25 °C on a shaker. Finally, 50 µL Griess solution B was added and the plate was incubated on a shaker for 10 min at 25 °C, before absorbance at 540 nm mas measured in a Tecan SPARK microplate reader (Tecan, Groedig, Austria). Nitrite/nitrate concentration was calculated with a nitrite + nitrate standard curve by subtracting the medium blank value from all wells. For total concentration of NO in the samples, the concentrations of NO^3−^ + NO^2−^ were summed up.

### 2.14. MMP2 and MMP9 in Gel Zymography

For the determination of MMP2 and MMP9 enzymatic activity, cell culture supernatants of 3D tendon-like constructs were analyzed by in gel gelatin zymography. Therefore, 10% SDS-polyacrylamide gels were co-polymerized with 1 mg/mL gelatin. The cell culture supernatants (30 µL each) were mixed with Tris-Glycine Laemmli SDS Sample Buffer and incubated for 10 min at RT, then samples were loaded and gel was run with 1× Tris-Glycine SDS-PAGE running buffer. For enzyme activity detection gels were incubated in 1× renaturing buffer (2.5% Triton X-100 (*v*/*v*) in water) for 30 min at RT with gentle shaking to remove SDS and allow MMPs to renature, followed by 30 min in 1× zymogram developing buffer (50 mM Tris-HCl pH7.45, 200 mM NaCl, 5 mM CaCl_2_, and 0.02% (*v*/*v*) Brij 35). The developing buffer was replaced once and gels were incubated over night at 37 °C with gentle agitation for maximal sensitivity. On the next day, the gels were stained for 30 min with 0.5% (*w*/*v*) Coomassie Brilliant Blue in 40% (*v*/*v*) ethanol and 10% (*v*/*v*) acetic acid in water and destained with a mixture of 40% (*v*/*v*) ethanol and 10% (*v*/*v*) acetic acid in water until the clear bands got visible. Those clear bands represented the areas, where the MMPs had digested the gelatin substrate. For quantification the gels were photographed with the ChemiDoc MP device (BioRad, Munich, Germany) and the band intensities were measured using the Volume tool of the software ImageLab (BioRad, Munich, Germany).

### 2.15. Statisical Analysis

Statistical analyses were performed using Graph Pad Prism software (version 5.04). Densitometric data are presented as means with standard deviations. One-way analysis of variation (ANOVA) applying the nonparametric KruskalWallis test were used to test for differences between the groups. Pairwise analysis of qRT-PCR data was performed using the Mann-Whitney test. A *p*-value < 0.05 was considered statistically significant.

## 3. Results

### 3.1. Experimental Design and RNASeq Data Analysis

**Figure 1:**
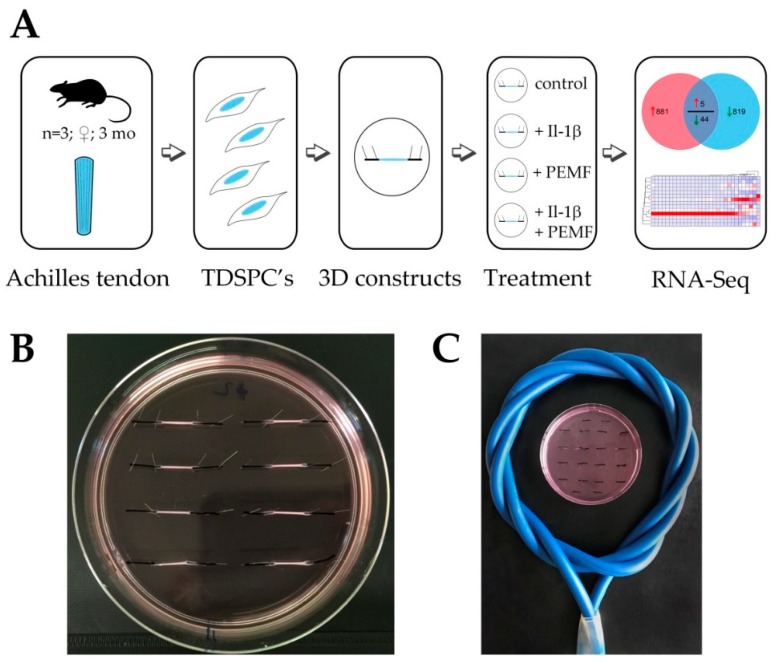
Experimental setup for high energy pulsed electromagnetic field (PEMF/rPMS) treatment of tendon-like constructs. (**A**) Tendon derived stem progenitor cells were isolated from 3 months old female F344 rats (*n* = 3) and used for the generation of 3D tendon-like constructs. Four different treatment groups were established: an untreated control group, a +Il-1β treated group, a +PEMF treatment group and a +PEMF+Il-1β treatment group. After PEMF treatment regimen total RNA of the 4 treatment groups was isolated for RNASeq analysis. (**B**) Image of tendon-like constructs after 7 days of contraction. (**C**) Position of tendon-like constructs in PEMF coil during treatment.

As outlined in the experimental design workflow (Figure 1A), total RNA samples were harvested from In vitro cultured 3D-embedded tenocyte cultures generated from TDSPCs isolated from rat Achilles tendons. In total, 3 biological replicates (tendon constructs of 3 individual animals) were obtained and 8 constructs per animal and treatment were assembled in a single dish (Figure 1B). Constructs were either stimulated with Il-1β for 12 h or left untreated and subsequently exposed to PEMF/rPMS as described in the methods section (Figure 1C). In total 12 RNA-seq libraries were prepared and sequenced at depths of 24.23 to 31.96 million reads per sample. Across all libraries, single-end read alignment rates to the Rnor_5.0 rat genome were between 84.05 and 92.98%. For differential analysis two pairwise comparisons were performed using Cuffdiff2: (1) untreated control vs. Il-1β stimulation (2) PEMF treatment vs. Il-1β stimulation and PEMF treatment (see supplementary Table S1).

**Figure 2:**
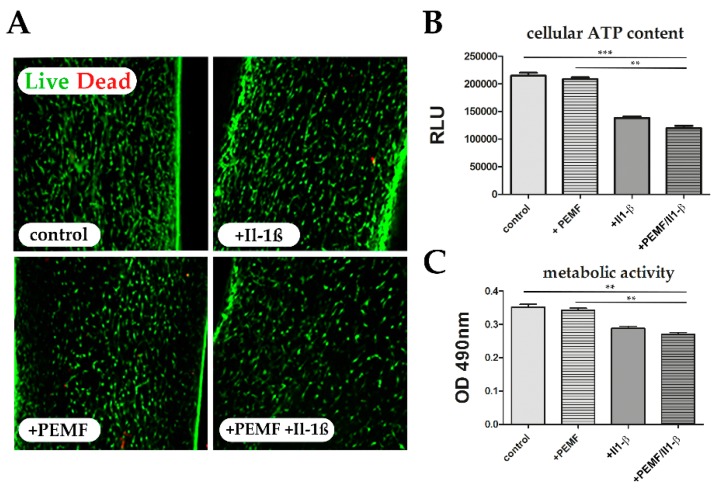
Cell viability and cellular metabolic activity of treated tendon-like constructs (**A**) Live/Dead Assay showing viable cells in green and dead cells in red. (**B**) Total cellular ATP content after +Il-1β and +PEMF+Il-1β treatment is significantly diminished compared to untreated control or +PEMF treatment alone. (**C**) MTT Assay was performed for determination of metabolic activity of TDSPCs, showing a significant decrease in metabolic activity in the +Il-1β and +PEMF+Il-1β treatment groups. ** *p* < 0.01, *** *p* < 0.001.

Importantly, neither stimulation with Il-1β nor the exposure to PEMF reduced the viability of the 3D-embedded constructs as evidenced by Live/Dead staining (Figure 2A). Further, PEMF exposure did not negatively impact the metabolic activity or ATP production of the embedded TDSPCs. Not surprisingly, 24 h stimulation with 10 ng/mL Il-1β moderately lowered the metabolic activity of the cells, with no differences evident after PEMF exposure (Figure 2B,C). Taken together, PEMF and Il-1β treatment of the 3D constructs did not elicit a significant cytotoxic effect. 

### 3.2. Global Responses in Il-1β-Primed TDSPCs after PEMF Exposure

**Figure 3:**
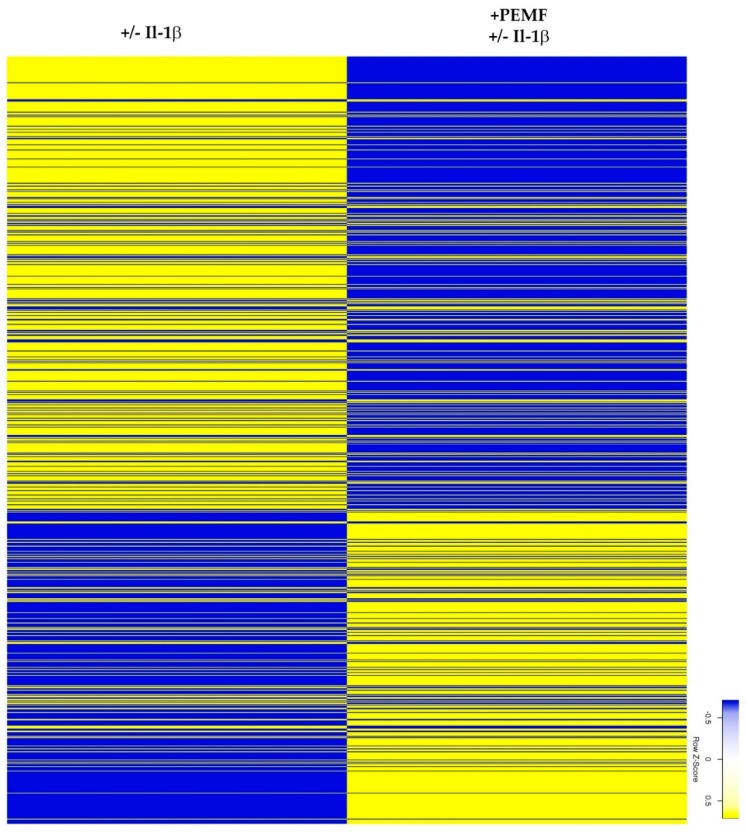
Unclustered heatmap of differentially expressed genes demonstrates global responses of 3D constructs after PEMF exposure (≥2.5-fold change; *p* value < 0.05).

Exploratory heatmap analysis revealed that the exposure of Il-1β-treated 3D tendon-like constructs resulted in a change in the expression pattern of approximately 5400 genes (*p* value < 0.05; Figure 3 and supplementary Table S1). 

In order to further characterize the genes regulated in response to PEMF, gene ontology (GO) analysis was performed based on genes identified to be differentially regulated between the 2 groups (fold change [+PEMF +/− Il-1β] vs. [+/− Il-1β]; Supplementary Table S1). The top 20 GO terms ranked by the corrected *p* value are listed in Table 1. Interestingly, next to more general terms (e.g., response to drug, regulation of cell proliferation, etc.), differentially expressed genes were assigned to processes driving the negative regulation of cell apoptosis, extracellular matrix and collagen fibril organization, and wound healing (see supplementary Table S2 for assigned genes). 

**Figure 4:**
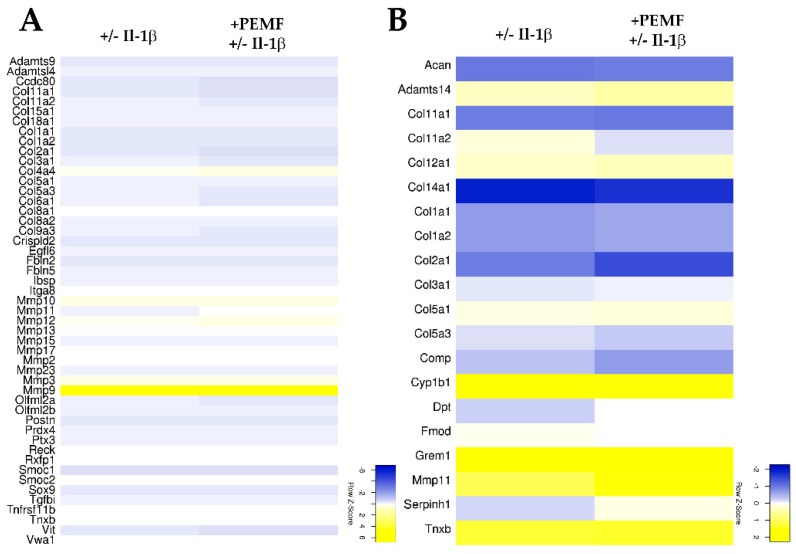
Heatmaps summarizing the differential expression of genes associated (**A**) with GO term “extracellular matrix organization” and (**B**) genes assigned to the GO term “collagen fibril organization” (≥2.0 fold change; *p* value < 0.05).

**Figure 5:**
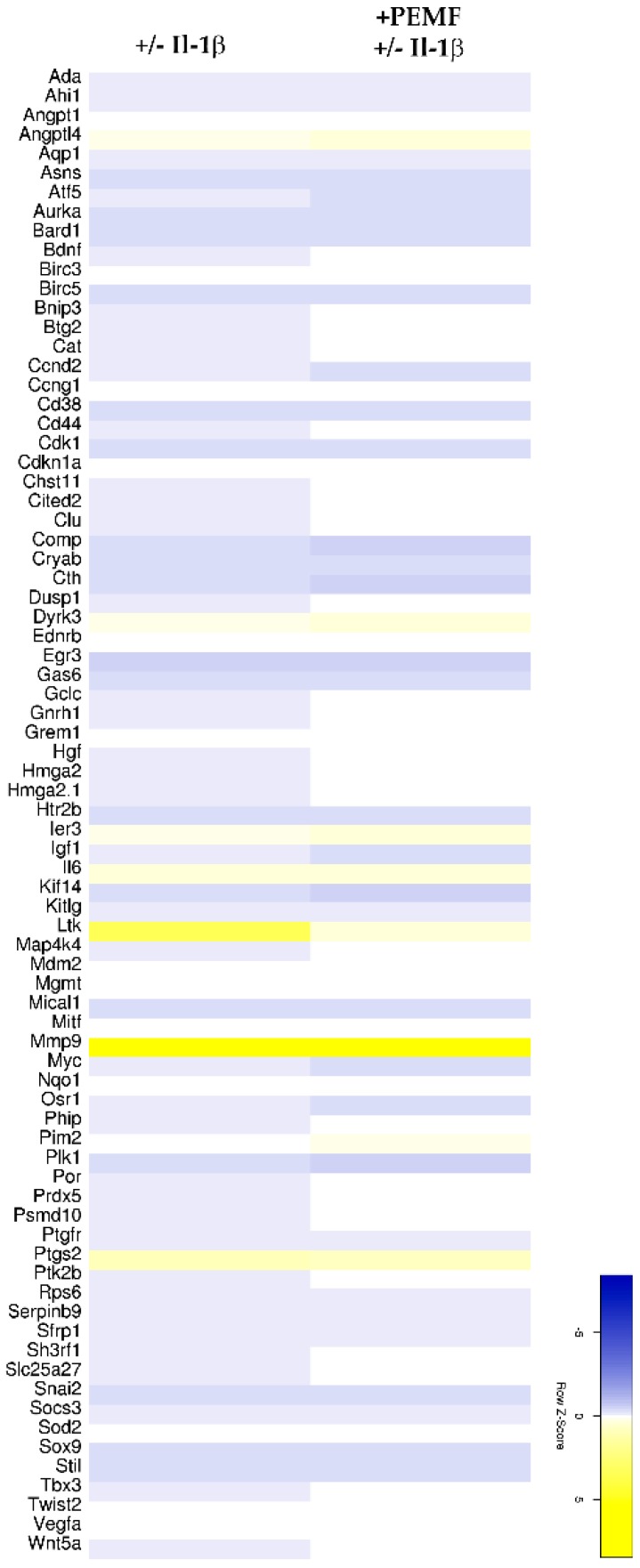
Heatmap of differentially expressed genes identified for the 2 paired groups ([−/+ Il-1β] and [+PEMF −/+ Il-1β]) assigned to the GO term “negative regulation of apoptotic process” (≥2.0 fold change; *p* value < 0.05).

Based on the assignment to the various biological processes, again an unclustered heatmap was generated for all genes showing a minimum 2-fold difference in expression. As shown in Figure 4A,B, for genes associated with remodeling of the extracellular matrix and collagen fibril organization only moderate differences were evident. In comparison, 77 genes associated with negative regulation of apoptosis showed a more robust response after PEMF exposure (Figure 5 and supplementary Table S3).

**Figure 6:**
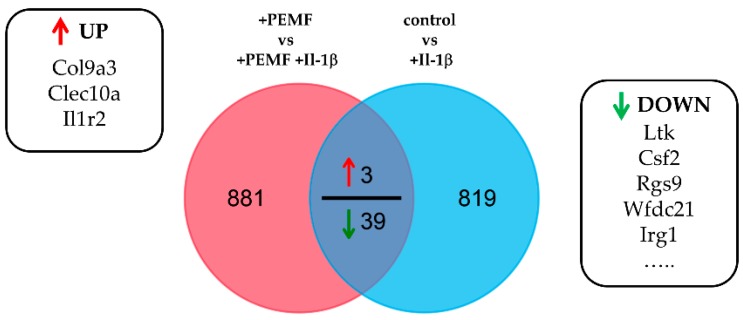
Quantitative VENN diagram showing overlap of genes derived from differential expression analysis. Commonly regulated genes (≥2.0 fold change) are partially shown in the boxes. 3 of these genes were significantly up-regulated, whereas 39 genes were identified to be down-regulated (top 5 shown).

Finally, we determined the commonly differentially expressed genes in response to PEMF exposure after Il-1β treatment. In other words, we determined how does PEMF exposure modulate the response of 3D tendon constructs to Il-1β stimulation. As demonstrated by Venn diagram analysis (Figure 6), only a small number of genes showed a response greater than 2-fold (*p* value < 0.05). 

Nevertheless, several of the genes identified belong to either extracellular matrix proteins (e.g., Col9a3), are involved in inflammation and/or cytoprotection (e.g., Il1r2, Csf3), or the regulation of apoptosis (Ltk), further underscoring the results of the GO analysis (see Table 2).

### 3.3. PEMF Exposure Does not Alter the Structure or Turn-Over of the Extracellular Matrix in 3D Cultures

**Figure 7:**
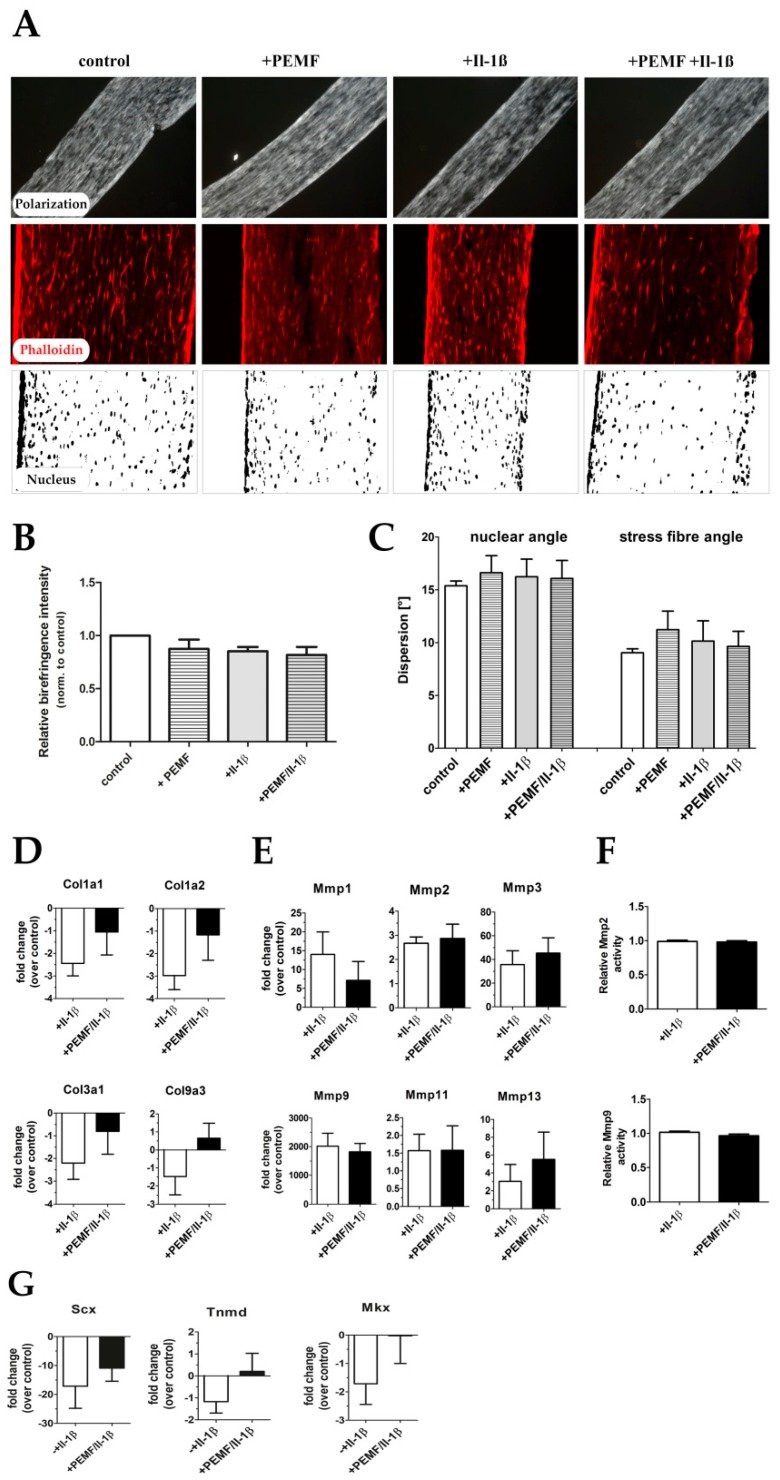
Extracellular matrix organization in tendon-like constructs before and after Il-1β and/or PEMF treatment. (**A**) Polarization microscopic images, phalloidin staining, and nuclear stain (DAPI) of 3D tendon constructs. (**B**) Relative birefringence intensities as surrogate for collagen fiber organization (*n* = 4). (**C**) Actin stress fiber angle and nuclear angle dispersion determined for the 4 treatment groups (*n* = 4). (**D**) The expression of extracellular matrix genes *Col1a1*, *Col1a2*, *Col3a1* and *Col9a3* is shows up-regulation PEMF treatment in pro-inflammatory stimulated (+PEMF +Il-1β) samples although not statistically significant (*n* = 9). (**E**) Under pro-inflammatory conditions the matrix metalloproteinases-1, -2, -3, -9, -11 and -13 show no significant changes in their expression after PEMF treatment (*n* = 7). (**F**) Relative MMP2 and MMP9 activity in Il-1β-stimulated constructs with and without PEMF exposure (*n* = 10). (**G**) Expression of tendon related marker genes Scleraxis (Scx), Tenomodulin (Tnmd), and Mohawk (Mkx) is restored after PEMF treatment of pro-inflammatory primed tendon constructs (*n* = 6).

As GO assignment indicated that genes involved in ECM remodeling and also collagen fibrillogenesis were affected, we next examined if PEMF exposure results in a structural change of the 3D tendon-like constructs. Therefore, constructs generated from a total of 4 individual rats were generated, while collagen alignment and packing were analyzed by polarization microscopy. The overall collagen fiber orientation and packing was not significantly altered in response to Il-1β treatment, PEMF treatment alone, or in combination respectively as evidenced by birefringence signal intensities (Figure 7A,B). Also, we did not observe any significant changes in nuclear angle and actin cytoskeleton arrangement, indicating that there was no significant impact on cellular alignment in the treated constructs (Figure 7A,C). We next examined the effect of PEMF exposure on the mRNA levels of several genes encoding ECM proteins and MMPs by qRT-PCR. As expected, treatment of the 3D-embedded TDSPC cultures with Il-1β resulted in a significant decrease in expression of type I and type III collagen (Figure 7D). Although by trend PEMF exposure did partially rescue collagen expression, none of the results were statistically significant, except for the fibril-associated type IX collagen, confirming the results from the differential gene expression analysis (see Table 2). Il-1β stimulation further significantly enhanced the expression of several matrix metalloproteases, but again PEMF treatment did not significantly alter their expression (Figure 7E) or the activity of MMP2 or MMP9 as evidenced by densitometric analysis of gelatin zymograms (Figure 7F). Taken together, 2 cycles of 90 min PEMF/rPMS treatment (82mT, 125kHz) do not elicit any acute, short-term structural ECM changes, nor does they significantly attenuate the induction of MMPs. Il-1β treatment decreased the expression of tendon-related genes like scleraxis, tenomodulin, and mohawk, PEMF exposure did partially restore the expression of these genes, although this was not statistically significant (Figure 7G). 

### 3.4. Treatment with PEMF Drives Expression of Cytoprotective Cytokines

**Figure 8:**
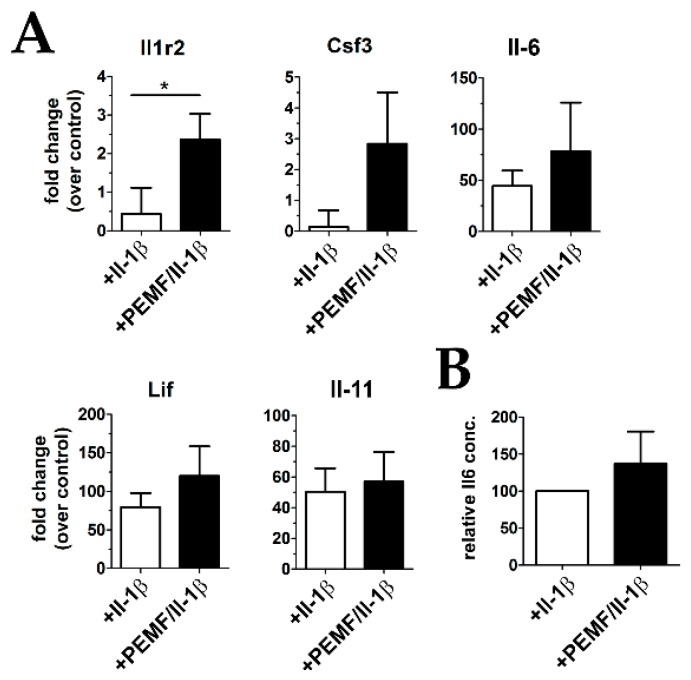
mRNA expression of cytoprotective cytokines. (**A**) Gene expression of Il1r2 was significantly up-regulated after PEMF treatment, and some members of potentially cytoprotective cytokines of the Il-6/gp130 family also show an increase by trend (*n* = 9) (**B**) Il-6 cytokine levels were also elevated in culture supernatants PEMF-treated constructs as evidenced by ELISA analysis (*n* = 5). (Lif leukemia inhibitory factor, Csf3 colony-stimulating factor 3). * *p* < 0.05.

As PEMF treatment has been shown to positively influence the expression and release of anti-inflammatory cytokines, we next verified the differential expression of the Il-1β decoy receptor Il1r2 which was identified as one of the top regulated genes by RNASeq analysis. In addition, the change in expression of the Il-6/gp130 family cytokines Il-6, colony-stimulating factor 3 (Csf3), leukemia inhibitory factor (Lif), and Il-11 was evaluated by RT-qPCR. As shown in Figure 8A, the expression of Il1r2 was increased around 5-fold after exposing Il-1β-primed constructs to PEMF. In addition, Csf3, Il-6, and Lif showed a moderate, but statistically not significant increase in mRNA expression, whereas no difference was evident for Il-11. On the protein level, secreted Il-6 levels were also found to be elevated (around 50%), however the responses of the individual biological replicates were very heterogenous. Nevertheless, in summary these results indicate that exposure to PEMF elicits a pleiotropic, anti-inflammatory response in part mediated by the expression of cytoprotective cyokines and dampening the pro-inflammatory action of Il-1β by elevating the levels of the decoy receptor Il1r2. 

### 3.5. PEMF limits Il-1β-Induced Apoptosis

**Figure 9:**
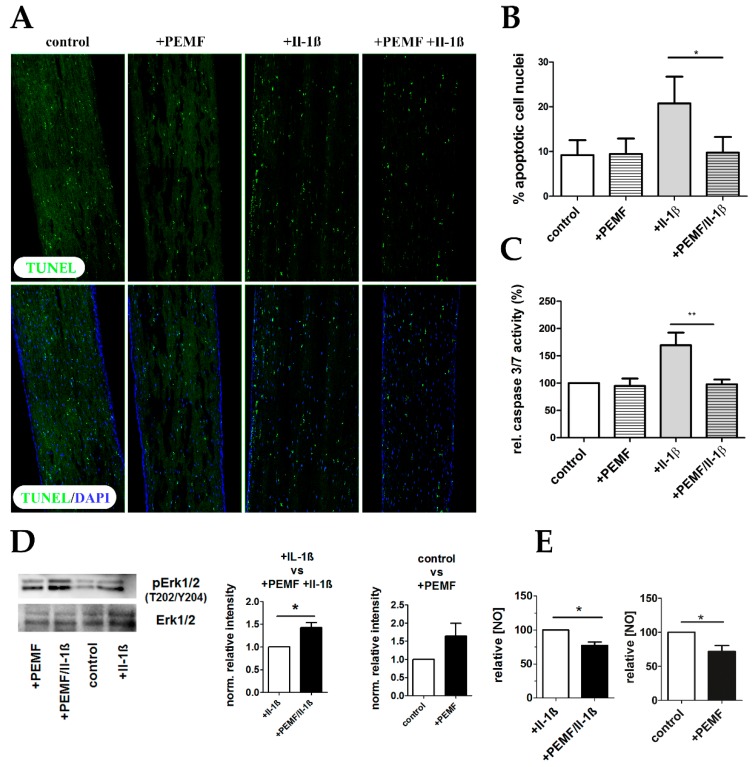
PEMF treatment attenuates apoptosis in pro-inflammatory stimulated tendon-like constructs. (**A**) TUNEL Assay was performed to visualize apoptotic cell nuclei (green) showing the characteristic fragmented phenotype. (**B**) Percent apoptotic cell nuclei was determined, demonstrating an anti-apoptotic effect of PEMF exposure of Il-1β-primed 3D constructs. (**C**) Caspase3/7 enzyme activity was determined in protein lysates of tendon-like constructs of all for treatment groups, confirming that PEMF treatment significantly decreases apoptosis in pro-inflammatory stimulated samples. (**D**) Representative Western blot and densitometric analysis (*n* = 4) for phosphoERK1/2 and totalERK1/2 demonstrate activation of ERK1/2 after PEMF exposure. (**E**) Total NO concentration is significantly reduced in cell culture supernatants of +PEMF +Il-1β and +PEMF treated tendon like constructs (*n* = 5). * *p* < 0.05, ** *p* < 0.01.

As a significant number of differentially regulated genes were assigned to the GO term “negative regulation of apoptosis”, we examined if PEMF-treatment indeed is able to attenuate Ιl-1β-mediated apoptosis. Priming of 3D–embedded TDSPCs with 10 ng/mL Il-1β resulted in a significant increase in apoptotic cells as determined by in situ detection of fragmented DNA (TUNEL assay; Figure 9A,B). PEMF-treatment significantly reduced the number of apoptotic cells and the anti-apoptotic effect was further underscored by baseline caspase 3 and 7 enzyme activities when compared to the constructs cultured under pro-inflammatory cell culture conditions (Figure 9C). As activation of Erk1/2 generally promotes cell survival, we next examined the levels of Erk1/2 phosphorylation after PEMF exposure. Indeed, PEMF-treatment generally increases pErk1/2 levels, not only under pro-inflammatory conditions (Figure 9D). Finally, as nitric oxide (NO) is well known to affect apoptosis, we determined the NO content in cell culture supernatants. As shown in Figure 9E, NO levels were significantly reduced in PEMF-treated 3D tendon constructs after Il-1β priming. In conclusion, PEMF treatment significantly reduces Il-1β-induced apoptosis in TDSPCs cultured in 3D. 

## 4. Discussion

The aim of this study was to investigate the global response of 3D tendon constructs to PEMF exposure under inflammatory conditions to gain mechanistic insight into the processes regulated by PEMF treatment, which is frequently used as a non-invasive physical therapy for tendinopathy. Tendinopathy is a multifactorial disorder accounting for a high number of patients consulting a general or orthopedic practitioner [1]. Currently, tendon disorders are either treated by a conservative approach (e.g., physiotherapy, extracorporeal shockwave therapy, anti-inflammatory drugs) or by surgical intervention, often resulting in unsatisfactory outcomes. Although the development of new and effective treatment strategies is limited by our incomplete understanding of the cellular and molecular processes leading to tendinopathies, pulsed electromagnetic field therapy is believed to have beneficial effects on tendon conditions.

Pulsed electromagnetic field (PEMF) therapy is a non-invasive, non-thermal treatment mostly applied to promote healing. The U.S. Food and Drug Administration approved PEMF therapy to treat non-union bone fractures, post-operative pain, edema, and osteoarthritis [30]. The biophysical mechanisms through which PEMF elicits cellular and molecular responses are complex and remain largely unresolved. It is hypothesized that the primary mechanism of action is to facilitate the reduction and resolution of inflammatory processes. For example, low frequency PEMF applied for 8 h on tendon-derived cells in a 2D culture system revealed, next to an increased tendon-related marker expression, the enhanced release of anti-inflammatory cytokines [31]. Pre-clinical studies on a rat rotator cuff repair model indicated that PEMF treatment improves early tendon healing and a concomitant increase in the Young’s modulus and maximum stress to failure of the tendon tissue [32]. In another preclinical study, PEMF increased the tensile strength of the healed Achilles tendon after full transection in a rat model [33]. Clinically, PEMF therapy was shown to have positive effects on rotator cuff tears affecting multiple pain parameters and improving the range of motion and muscle strength [34], indicating a positive effect on tendon healing. However, as the causal cellular and molecular mechanisms for PEMF action on tendon-resident cells are insufficiently understood, we aimed to explore the global response to PEMF exposure in inflammatory-primed 3D tendon like constructs by performing RNA sequencing. RNA sequencing identified 5400 genes to be differentially expressed upon PEMF exposure under Il-1β stimulation, of which several are known to regulate apoptosis, extracellular matrix organization or collagen fibril organization, and genes encoding for cytoprotective proteins.

The pathophysiological mechanisms of tendinopathy so far described include dysregulated apoptosis, mechanical overload, matrix metalloproteinase activation, genetic disposition, and inflammation. In the past years evidence for the role of inflammation in the development of tendinopathy has grown, although the key players remain to be fully characterized. Interleukin-1β levels are known to be increased in tendinopathy and following tendon injury. Several studies have shown that Il-1β stimulation of tendon-derived cells represses collagen type 1 expression, increases the expression of matrix metalloproteinases [5,7,35,36] or leads to cytotoxic effects and caspase activation both in 2D and 3D cell cultures [37,38]. It also was shown that Il-1β irreversibly downregulates the expression of tenogenic makers such as scleraxis or tenomodulin and alters cell metabolism in tendon cells isolated from injured tendons [36]. As evidenced by qPCR analysis, in our study PEMF exposure partially restored the expression of collagen genes and the tenogenic markers Tnmd and Mkx after pro-inflammatory stimulation. Further, a significant increase in MMP expression upon Il-1β stimulation was seen, which is in agreement with previously published data. The major matrix metalloproteinases promoting tendon damage and degeneration are MMP1, MMP2, MMP3, MMP9, and MMP13, where MMP1 and MMP13 are predominantly involved in the degradation of collagens type 1, type 2 and type 3. Subsequently MMP2 and MMP9 proteolytically degrade these collagenous fragments to smaller entities. MMP3 is involved in the proteolytic activation of other MMPs and MMP3 together with MMP2 can promote the healing process. An increased net MMP activity is supposed to be an indicator for matrix degradation, which might be an early part of matrix remodeling in wound healing [35]. However, we did not see a significant impact on MMP expression or activation upon PEMF exposure. This is also in agreement with a previous study by Ongaro A. et al., demonstrating that low-intensity PEMF did not affect matrix degrading enzyme production, but had anti-inflammatory effects in synovial fibroblasts isolated from osteoarthritis patients [39].

The pleiotropic cytokine Il-6 has a central role in inflammation and tissue injury and it was shown to be enhanced in tendon-resident cells after Il-1β treatment, inducing the acute response phase and enhancing the healing phase by promoting collagen type 1 expression [35]. Interestingly, extracorporeal shockwave therapy (ESWT) applied to tendon cells increased Il-6 expression [40] and recombinant Il-6 was used as a therapeutic intervention by infusion into the peritendinous tissue of human Achilles tendons, resulting in increased collagen synthesis [41]. Additionally, Il-6 was shown to have an inhibitory effect on the expression of complement regulatory proteins, suggesting that Il-6 can reduce the sensitivity of tenocytes to complement-mediated cell lysis [7]. Here we show that two exposures of 60 min PEMF/rPMS at 82mT positively influenced the expression and the release Il-6 in inflammatory stimulated tendon-like constructs. In addition, genes encoding the cytoprotective proteins Csf3, or Lif were moderately enhanced after PEMF exposure. Interestingly, the Il-1β decoy receptor Il1r2 was significantly higher expressed after PEMF exposure, most likely dampening the pro-inflammatory action of Il-1β. Il1r2 recently has been shown to play a central role in the protection of embryonic stem cells- (ESC-) derived tenocytes from Il-1β-mediated inflammation in 3D tendon cultures. McClellan et al. demonstrate that Il-1β diminishes the ability of fetal and adult tenocytes to form mature tendon-like constructs, whereas ESC-derived constructs appeared normal. As ESCs-derived tenocytes expressed high levels of Il1r2 the translocation of NF-κB into the nucleus after Il-1β stimulation was significantly reduced, possibly conferring cytoprotection [37].

Nitric oxide (NO) is a key molecule in the pathogenesis of inflammation and is produced upon tendon injury. Nitric oxide synthases (NOS) are upregulated in tendinopathy [5,42] impacting on the expression of several cytokines and on collagen synthesis. In connective tissue cells NO contributes to apoptosis under inflammatory conditions and elevated NOS levels were shown to be associated with apoptosis in Achilles tendinopathy [7]. Besides enhanced NO levels, elevated caspase-3 activity in inflamed equine tendons has also been observed and it is believed that increased cell death by apoptosis and an impaired clearance of apoptotic cells affects tendon homeostasis and contributes to tendon degeneration. Further, Erk1/2 activation has been shown to promote cell survival by driving anti-apoptotic processes by a wide range of responses involving either transient or prolonged activation, whereas Erk1/2 inhibition is known to have a pro-apoptotic effect [43]. However, the mechanisms by which Erk1/2 activation controls apoptosis are complex and vary depending on the cell or tissue type studied. Erk1/2 can promote cell survival either by suppressing the function of pro-apoptotic proteins and/or by enhancing the activity of anti-apoptotic molecules (e.g., regulation of anti-apoptotic transcription factors, up-regulation of the translation of anti-apoptotic proteins) [44]. Overall, although the global gene expression responses were generally moderate, the multimodal action of PEMF-treatment resulted in a significant reduction of apoptosis in 3D-embedded rat tenocyte cultures.

Taken together, our results suggest that high energy PEMF limits the catabolic effects of a pro-inflammatory stimulus by Il-1β by inducing the expression of cell protective molecules and attenuating apoptosis, thereby shifting a degenerative, inflammatory milieu to a more tissue reparative state.

## Figures and Tables

**Table 1:** Top 20 GO terms assigned to differentially expressed genes in Il-1β-stimulated tendon constructs after PEMF exposure.

Biological Process	% Genes	Fold Enrichment	Corrected *p* Value
response to hypoxia	3.044	2.032	6.18 × 10^−12^
negative regulation of apoptotic process	4.959	1.698	3.06 × 10^−11^
positive regulation of gene expression	4.330	1.765	3.93 × 10^−11^
response to drug	4.776	1.708	5.34 × 10^−11^
cellular response to tumor necrosis factor	1.942	2.293	7.95 × 10^−10^
extracellular matrix organization	1.837	2.350	8.19 × 10^−10^
response to estradiol stimulus	2.362	2.092	1.55 × 10^−9^
wound healing	1.679	2.398	2.92 × 10^−9^
response to lipopolysaccharide	2.729	1.949	5.02 × 10^−10^
positive regulation of cell proliferation	4.907	1.606	1.64 × 10^−8^
negative regulation of gene expression	2.650	1.907	5.10 × 10^−8^
in utero embryonic development	2.755	1.853	1.57 × 10^−7^
Aging	3.201	1.755	2.4 × 10^−7^
cytoplasmic translation	0.787	3.223	4.20 × 10^−7^
positive regulation of cell migration	2.467	1.893	4.53 × 10^−7^
protein transport	2.309	1.925	6.84 × 10^−7^
regulation of cell proliferation	2.020	2.012	8.89 × 10^−7^
apoptotic process	3.385	1.690	1.25 × 10^−6^
collagen fibril organization	0.813	3.059	1.48 × 10^−6^
negative regulation of cell proliferation	3.437	1.667	2.58 × 10^−7^

**Table 2:** Summary of common gene responses comparing 3D tendon constructs treated with Il-1β alone (+/− Il-1β) or exposed to PEMF under Il-1β (+PEMF +/− Il-1β).

Gene Symbol	Gene Name	Fold Change	Gene Symbol	Gene Name	Fold Change
*Ltk*	Tyrosine-protein kinase receptor	−10.36	*Irg1*	Immune-Responsive Gene 1 Protein	−2.45
*Csf2*	Granulocyte-macrophage colony-stimulating factor	−10.16	*Ereg*	Proepiregulin	−2.44
*Rgs9*	Regulator of G-protein signaling 9	−4.61	*Egr3*	Early growth response protein 3	−2.43
*Wfdc21*	WAP four-disulfide core domain 21	−4.41	*Fam84a*	Family with sequence similarity 84, member A	−2.41
*Itga7*	Integrin alpha-7	−3.86	*Cited1*	Melanocyte-Specific Protein 1	−2.29
*Scgb1c1*	Secretoglobin family 1C member 1	−3.80	*Csf3*	Colony-stimulating factor 3	−2.28
*Fam71e2*	Family with sequence similarity 71, member E2	−3.60	*Cnn1*	Calponin-1	−2.27
*Adgb*	Androglobin	−3.59	*Gdf15*	Growth/differentiation factor 15	−2.16
*Cd40*	CD40 molecule	−3.56	*Ptgs2*	Prostaglandin G/H synthase 2	−2.15
*Car12*	Carbonic anhydrase 12	−3.51	*Actg2*	Actin, gamma-enteric smooth muscle	−2.14
*Cxcl2*	C-X-C motif chemokine 2	−3.40	*Scube1*	Signal Peptide, CUB Domain, EGF-Like 1	−2.14
*Ip6k3*	Kinase	−3.21	*Pvalb*	Parvalbumin alpha	−2.14
*Rdh5*	Retinol dehydrogenase 5	−3.05	*Ggt1*	Glutathione hydrolase 1 proenzyme	−2.13
*Nsg2*	Neuronal vesicle trafficking-associated protein 2	−2.84	*Igsf9b*	Protein turtle homolog B	−2.09
*Fam64a*	Family with sequence similarity 64, member A	−2.84	*Myb*	MYB proto-oncogene, transcription factor	−2.07
*Ass1*	Argininosuccinate synthase	−2.78	*Dlgap2*	Disks large-associated protein 2	−2.04
*Prom1*	Prominin 1	−2.61	*Neurl3*	E3 ubiquitin-protein ligase NEURL3	−2.03
*RGD1561143*	Similar to cell surface receptor FDFACT	−2.54	*Col9a3*	Collagen type IX alpha 3 chain	2.05
*Ces1a*	Carboxylic ester hydrolase	−2.49	*Clec10a*	C-type lectin domain family 10 member A	2.05
*Fbn2*		−2.47	*Il1r2*	Interleukin-1 receptor type 2	2.78

## Supplementary Materials

The following are available online at https://www.mdpi.com/2073-4409/8/5/399/s1, Table S1: RNASeq data; Table S2: GO term-assigned genes; Table S3: List of genes assigned to GO terms *negative regulation of apoptotic* process, *ECM remodeling* and *collagen fibril organization*; Table S4: Oligo and probe sequences of TaqMan Assays used for gene expression analysis.
