# Supplementary material for: Global Responses of Il-1β-Primed 3D Tendon Constructs to Treatment with Pulsed Electromagnetic Fields
Source: Cells. 2019 Apr 30;8(5):399. doi: 10.3390/cells8050399 (PMC6562657; doi:10.3390/cells8050399)
Supplement: Supplementary file 1 [file cells-08-00399-s001.zip › S2.docx]

| **GO term** | | **genes** |
| --- | --- | --- |
| negative reg. of apoptotic process | Ltk; Egr3; Ptgs2; Il6; Nqo1; Mmp9; Cryab; Ier3; Slc25a27; Sox9; Htr2b; Snai2; Phip; Fhl2; Mgmt; Bnip3; Grem1; Cdkn1a; Ccnd2; Dyrk3; Mdm2; Ccng1; Hspb1; Sfrp1; Rps6; Ednra; Apc; Sod2; Mitf; Vegfa; Ptgfr; Socs3; Ahi1; Higd1a; Smo; Atf5; Asns; Ung; Rnf144b; Cbl; Grk5; Rarg; Opa1; Sphk1; Angpt1; Ncoa3; Btg2; Kitlg; Braf; Plk1; Angptl4; Bcl3; Cd44; Ago4; Wnt5a; Bcl6; Smad3; Ada; Serpinb9; Rhbdd1; Ctsh; Ercc5; Gas6; Mical1; Anp32b; Flna; Gclc; Gnrh1; Pten; Chst11; Npm1; Ilk; Cd59; Tp53; Ptma; Bnip3l; Ptk2b; Rara; Map4k4; Igf1r; Dusp1; Rb1cc1; Pik3r1; Prdx5; Arel1; Sh3rf1; Pim2; Tgfbr1; Aqp1; Sirt1; Birc2; Ednrb; Raf1; Bard1; Hk1; Hypk; Rpl10; Cth; Thbs1; Tgfb2; Adam17; Eif5a; Pa2g4; Cdkn1b; Armc10; Xbp1; Ncl; Sgk1; Por; Psen1; Aatf; Cat; Osr1; Hspa5; Pcnt; Plk2; Akr1b1; Hdac2; Cdk1; Tbx3; Casp3; Bfar; Rps3a; Nme2; Ybx1; Ppt1; Stil; Glo1; Arf4; Araf; Ppif; Fas; Pink1; Naa35; Myc; Bcl2l1; Hip1r; Taf9b; Hspd1; Cflar; Twist2; Il7; Six1; Nuak2; Hspa4; Rnf157; Siah2; Birc3; Hand2; Ybx3; Bcl2l2; Prnp; Mad2l1; Bag3; Cited2; Stat5a; Axl; Plaur; Ddrgk1; Arhgap10; Fkbp8; Cln3; Sod1; Pdpn; Mif; Nfkb1; Aldh2; Aurka; Tmbim4; Ube2b; Gfer; Birc5; Psmd10; Il1rn; Clu; Hmga2; Cib1; Hgf; Pdcd4; Cd38; Pdgfrb; Gpx1; Kif14; Pidd1; Arrb1; Bdnf; Comp; Igf1; Amigo2; |  |
| extracellular matrix organization | Mmp10; Mmp3; Col4a4; Mmp9; Smoc1; Adamts9; Sox9; Col18a1; Eln; Egfl6; Nf1; Postn; Mmp23; Tnfrsf11b; Mmp13; Itga8; Prdx4; Kazald1; Smoc2; Bcl3; Col8a2; Tnxb; Fbln2; Fbln5; Col1a1; Nid1; Col1a2; Col6a1; Pdgfra; Dnajb6; Tgfb2; Olfml2b; Vwa1; Mmp2; Ccdc80; Col16a1; Reck; Rxfp1; Aplp2; Col3a1; Ibsp; Fbln1; Adamtsl4; Vit; Mmp16; Emilin1; Col11a1; Hsd17b12; Tgfbi; Ptx3; App; Col5a1; Col17a1; Col8a1; Mmp15; Hspg2; Col5a2; Mmp17; Pxdn; Crispld2; Col15a1; Col27a1; Csgalnact1; Olfml2a; Col5a3; Col2a1; Mmp12; Col11a2; Mmp11; Col9a3; |  |
| wound healing | Cxcl2; Il6; Mmp3; Nos2; Igf2; Ccl20; Fgfr2; Cx3cl1; Nbeal2; Notch2; Dcbld2; Itga2; Nf1; Fgf10; Dst; Postn; Fgf7; Vegfa; Tpm1; Notch4; Tgfb3; Palld; Plat; Arhgef19; Wnt5a; Ltbp1; Nrg1; Sdc2; Slc11a1; Tp53; Aqp1; Col1a1; Macf1; Pdgfra; Sdc1; Tgfb2; Il1a; Cav1; Ppl; Casp3; RGD735065; Pak1; Eng; Col3a1; P2ry2; Sparc; Elk3; Dcn; Optn; Plec; Fntb; Cdh3; Mif; Wnt5b; Fgfr1; Itga9; Gsn; Pdgfa; Il1b; Fmod; Egr1; Pdgfrb; Jag1; Mmp12; |  |
| apoptotic process | Nsg1; Egln3; Phlda1; Tnfrsf21; Bnip3; Tmem173; Plekhf1; Grem1; Zmat3; Cst3; Lmnb1; Plscr3; Plscr1; Nradd; Gdf6; Ep300; Madd; Grk5; Tnfrsf11b; Opa1; Zc3h12a; Mcm2; Tnfrsf9; Rb1; Tp53inp1; Rock1; Ctsc; Rhbdd1; Gas6; Casp4; Rmdn3; Sra1; Asah2; Tp53; Bak1; Chac1; Sh3kbp1; Sgms1; Prdx5; Efna5; Sh3rf1; Mapk1; Ogt; Aifm1; Tgfbr1; Rassf5; Kremen1; Ei24; Sp110; Bok; Sirt1; Fis1; Gulp1; Birc2; Hip1; Shisa5; Bax; Mef2c; Mef2d; Dap; Eif5a; Bnip1; Ahr; Xbp1; Rtkn; Sgk1; Aatf; Casp12; Rtn3; C1qbp; Usp53; Pdcl3; Cdk1; Gapdh; Chek1; Aimp2; Casp3; Bfar; Rps3; Zfp36l1; Casp7; Pak1; Fas; Aldoc; Cdk5; Ppp1r13b; Bcl2l1; Hip1r; Unc5c; Cflar; Tmem214; Inpp5d; Six1; Prkcd; Nuak2; Adamtsl4; Siah2; Aktip; Birc3; Bcl2l2; Rffl; Melk; Kank2; Ppp1r15a; Dram2; Fkbp8; Adam15; Ntn1; Chek2; Sqstm1; Map2k7; Relt; Cdca7; Unc5b; Birc5; Psmd10; Ifi27; Gsn; Nr4a1; Shc4; Cib1; Pdcd4; Gpx1; Pidd1; Arrb1; Prune2; Comp; Arg2; Ccl6; |  |
| collagen fibril organization | Cyp1b1; Adamts14; Serpinh1; Grem1; Loxl4; Nf1; Dpt; Tnxb; Ddr2; Adamts2; Col14a1; Loxl3; Tgfbr1; Col1a1; Col1a2; Col12a1; Tgfb2; Plod3; Acan; Col3a1; P4ha1; Col11a1; Col5a1; Col5a2; Foxc2; Fmod; Col5a3; Col2a1; Comp; Col11a2; Mmp11; |  |
