# Supplementary material for: Global Responses of Il-1β-Primed 3D Tendon Constructs to Treatment with Pulsed Electromagnetic Fields
Source: Cells. 2019 Apr 30;8(5):399. doi: 10.3390/cells8050399 (PMC6562657; doi:10.3390/cells8050399)
Supplement: Supplementary file 1 [file cells-08-00399-s001.zip › S4.docx]

**Table S4: Oligo and probe sequences of TaqMan Assays used for gene expression analysis**

| **Gene name** | **Assay ID** | **oligo1 (5´-3´)** | **oligo2 (5´-3´)** | **probe (5´-3´)** | |
| --- | --- | --- | --- | --- | --- |
| **Col1a1** | Rn.PT.58.8986002 | GAGAACCAGCAGAGCCA | GAACAAGGTGACAGAGGCATA | /56-FAM/TGGAGACCA/ZEN/GAGAAGCCACGATGA/3IABkFQ/ | |
| **Col1a2** | Rn.PT.58.36132987 | GTTCACCCTTCACACCCT | GAGTCGTTGGGCCACAA | /56-FAM/ACCGTTGTG/ZEN/TCCTCGAATGCCTTT/3IABkFQ/ | |
| **Col3a1** | Rn.PT.58.35133758 | CCTGGATTACCATTGTTGCC | CCCTGGTGGTTCTGGAC | /56-FAM/ACTGCCCCG/ZEN/TTCACCCTTTACA/3IABkFQ/ | |
| **Col9a3** | Rn.PT.58.45203566 | TTGCCGACTTCTCCTTGTTC | CTGAAGGTGCTACTGATCTGC | /56-FAM/TGTCCTGCC/ZEN/ATCTGCCCTCC/3IABkFQ/ | |
| **Csf3** | Rn.PT.58.37548055 | CTTTGTCTGCTGCAAGGC | GGAACACTGAACTGCTGGA | /56-FAM/CCTCCGGAT/ZEN/GACACAGCTTGTAGG/3IABkFQ/ | |
| **Il1r2** | Rn00588589_m1 | no sequence information available | | |  |
| **Il11** | Rn.PT.58.45264338 | CCTAGGATGGCATGAGCTG | CGACTGGAACGGCTACTTC | /56-FAM/TAGGCGAGA/ZEN/CATCAAGAGCTGTAAGC/3IABkFQ/ | |
| **Il6** | Rn.PT.58.13840513 | CCTTCTGTGACTCTAACTTCTCC | CAGAGCAATACTGAAACCCTAGT | /56-FAM/TCCTTCCTA/ZEN/CCCCAACTTCCAATGC/3IABkFQ/ | |
| **Lif** | Rn.PT.58.36425014 | GCACATAGCTTATCCACGTTG | CAACCTCATGAACCAGATCAAG | /56-FAM/CAGTGCCAA/ZEN/TGCCCTCTTTATTTCCT/3IABkFQ/ | |
| **Mmp1** | Rn.PT.58.7424580 | TCATGAGCCGTAACATAGAACA | GAC TTG CTC ACA CAT TCC CA | /56-FAM/ACG TGG ACC /ZEN/GAC AAC AGT GAC AA/3IABkFQ/ | |
| **Mmp11** | Rn.PT.58.10854331 | ACTTTCCAGGACTTTCACCTTC | CCTTCTGAGATTGATGCTGCT | /56-FAM/TGCTGAGGG/ZEN/CTATGCCTACTTCC/3IABkFQ/ | |
| **Mmp2** | Rn.PT.58.8937436 | GAACACAGCCTTCTCTTCCT | GTT TAT TTG GCG GAC AGT GAC | /56-FAM/TGA CAA GCC /ZEN/CAC AGG TCC CTT /3IABkFQ/ | |
| **Mmp3** | Rn.PT.58.44652574 | CTGTGGAGGACTTGTAGACTG | CTATTCCTGGTTGCTGCTCAT | /56-FAM/AGCATTGGC/ZEN/TGAGTGAAAGAGACCC/3IABkFQ/ | |
| **Mmp9** | Rn.PT.58.7383134 | GGA GGT CAT AGG TCA CGT AGG | GAA CTC ACA CAA CGT CTT TCA C | /56-FAM/ACT CAC ACG /ZEN/CCA GAA GTA TTT GTC ATG G/3IABkFQ/ | |
| **Mmp13** | Rn.PT.58.9488961 | CCCTCCATAATGTCATACCCAT | CCAGAACTTCCCAACCATGT | /56-FAM/CATACGAGC/ZEN/ATCCATCCCGAGACC/3IABkFQ/ | |
|  |  |  |  |  | |
